# Disaster Preparedness Disparities: The Impact of Self-Efficacy, Information, Risk Perception Among Older Adults

**DOI:** 10.1093/geroni/igaf122.1976

**Published:** 2025-12-31

**Authors:** Yanjun Dong

**Affiliations:** SUNY at Albany, Albany, New York, United States

## Abstract

**Background:**

Disaster preparedness is essential for mitigating risks among older adults, yet disparities persist in how individuals prepare based on prior disaster experience and psychosocial factors. This study examines how experiencing a natural disaster influences preparedness actions through self-efficacy, information access, and risk perception, with a focus on racial differences between BIPOC and non-BIPOC older adults.

**Method:**

Using FEMA’s 2023 National Household Survey data (N = 2,225), we employ mediation analysis to assess both direct and indirect pathways linking disaster experience to preparedness behaviors among individuals aged 60 and older.

**Results:**

Findings indicate that disaster experience enhances preparedness both directly and indirectly. Self-efficacy (BIPOC: β = 0.11, p < 0.01; non-BIPOC: β = 0.15, p < 0.001) and information access (BIPOC: β = 0.07, p < 0.05; non-BIPOC: β = 0.10, p < 0.001) mediate this relationship, underscoring the role of confidence and information-seeking behavior in disaster readiness. While influenced by disaster experience, risk perception does not predict preparedness, suggesting that knowledge and self-efficacy are more critical drivers. Total effects show that BIPOC older adults (β = 0.69, p < 0.001) exhibit a stronger overall relationship between disaster experience and preparedness than their non-BIPOC counterparts (β = 0.62, p < 0.001), primarily due to a stronger direct effect.

**Discussion:**

These findings highlight the importance of enhancing self-efficacy and improving access to preparedness information, particularly for BIPOC older adults. Disaster preparedness programs should incorporate culturally tailored strategies to reduce disparities and ensure equitable resilience across diverse aging populations.

